# Dietary patterns are influenced by socio-demographic conditions of women in childbearing age: a cohort study of pregnant women

**DOI:** 10.1186/s12889-018-5184-4

**Published:** 2018-03-01

**Authors:** Juliana Araujo Teixeira, Teresa Gontijo Castro, Cameron C. Grant, Clare R. Wall, Ana Lúcia da Silva Castro, Rossana Pulcineli Vieira Francisco, Sandra Elisabete Vieira, Silvia Regina Dias Medici Saldiva, Dirce Maria Marchioni

**Affiliations:** 1 Department of Nutrition, School of Public Health, University of Sao Paulo, Sao Paulo, 01246-904 Brazil; 2 The Centre for Longitudinal Research – He Ara ki Mua, University of Auckland, Auckland, 1072 New Zealand; 3 Department of Paediatrics: Child and Youth Health, University of Auckland, Auckland, 1142 New Zealand; 4 Starship Children’s Hospital, Auckland District Health Board, Auckland, 1023 New Zealand; 5 Discipline of Nutrition and Dietetics, School of Medical Sciences, University of Auckland, Auckland, 1023 New Zealand; 6 Department of Obstetrics and Gynecology, School of Medicine, University of Sao Paulo, Sao Paulo, 05403-000 Brazil; 7 Department of Pediatrics, School of Medicine, University of Sao Paulo, Sao Paulo, 05403-000 Brazil; 8 Department of Health, Health Institute of Sao Paulo State, Sao Paulo, 01314-000 Brazil

**Keywords:** Dietary pattern, Principal component analysis, Socioeconomic factors, Childbearing age, Epidemiology, Public health nutrition

## Abstract

**Background:**

Women’s health during their reproductive years and whilst pregnant has implications for their children’s health, both in utero and during childhood. Associations of women’s pre-pregnancy dietary patterns (DP) with maternal socio-demographic characteristics and nutrient intake were investigated in ProcriAr cohort study in São Paulo/Brazil, 2012.

**Methods:**

The DPs of 454 women were investigated by principal component factor analysis, using dietary information from a validated 110-item food frequency questionnaire. Multiple linear regression models identified independent associations between DPs and maternal socio-demographic characteristics and Spearman’s correlation determined associations between DPs and nutrients intake.

**Results:**

Participants’ mean age was 26.1 years (standard deviation = 6.3), 10.3% had more than 8 years of formal education, 30% were migrants from outside of the Southeast of Brazil, 48% were employed, 13% were smokers, and 51% were overweight/obese. Four DPs were derived: ‘Lentils, whole grains and soups,’ ‘Snacks, sandwiches, sweets and soft drinks,’ ‘Seasoned vegetables and lean meats,’ and ‘Sweetened juices, bread and butter, rice and beans’. The ‘Lentils, whole grains and soups’ score was positively related to maternal age, being non-smoker and born in the South, North or Midwest of Brazil. The ‘Snacks, sandwiches, sweets and soft drinks’ score was positively related to higher maternal education, and negatively related to age, lack of formal work and being born in the Northeast region. The ‘Seasoned vegetables and lean meats’ score was positively related to higher maternal education. The ‘Sweetened juices, bread and butter, rice and beans’ score was positively related to unemployment and to no family history of hypertension, and negatively related to maternal overweight and obesity. Dietary intake of fruits and vegetables, foods that require preparation, nutrients from one-carbon metabolism, protein, iron, calcium and vitamin D were correlated with the ‘Seasoned vegetables and lean meats’. Dietary intake of sugar-sweetened and alcoholic beverages, industrialized and takeaway foods, and foods rich in sugar, energy, fat, and synthetic folate were correlated with the ‘Snacks, sandwiches, sweets and soft drinks’.

**Conclusions:**

Findings from this study add perspectives to be considered in the implementation of health interventions, which could improve women’s nutritional status and provide an adequate environment for the developing fetus.

**Electronic supplementary material:**

The online version of this article (10.1186/s12889-018-5184-4) contains supplementary material, which is available to authorized users.

## Background

Women’s health during the reproductive years and particularly during pregnancy is an important determinant of their children’s health, both in utero and during childhood [1]. Inadequate maternal nutrition during pregnancy is associated with reduced fetal growth and increased risk of respiratory disease in early childhood and then, later in life, cardiovascular diseases, type 2 diabetes, obesity and osteoporosis [2–6]. A focus on nutrition as a component of preconception care is recognized as essential if this care is to promote the health of the mother and to optimize fetal development. Thus, it is necessary to understand how social, demographic and behavioral factors of women of childbearing age can influence their broader dietary intake patterns as well as their intake of specific foods and nutrients [7]. Dietary patterns can be derived by using different methods, including numerical indexes aimed to measure adherence to specific patterns (e.g., Mediterranean Diet, Healthy Eating Index, or a nutritional guideline) or data-driven methods that use mathematics to empirically derive dietary patterns within the study population (e.g., cluster or factor analysis) [8]. Principal component factor analysis takes into account the cumulative and interactive diets’ aspects and, thus, generates data that better reproduce the actual dietary consumption rather than the description of specific foods and nutrients intake [8]. This method reflects food components interactions and improves the capacity to investigate the effects of diet on health [8].

To date, most of the nutritional recommendations concerning healthy pregnancy outcomes relate to specific foods, food groups or nutrients, making it difficult to translate appropriate dietary advice for non-dietitian health care professionals and for the women to whom advice is being given [9]. There is a need for nutritional interventions based on patterns of dietary intake but currently there is a lack of studies in developing countries that used this holistic approach. The aim of this study was to identify the dietary patterns of pregnant women from ProcriAr study during the pre-pregnancy period using principal component factor analysis, and to investigate the socio-demographic factors and nutrients associated with these patterns.

## Methods

### The ProcriAr cohort study

**Fig. 1:**
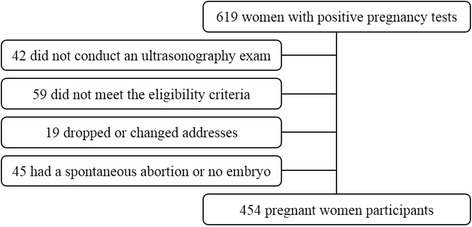
Description of the sample selection, ProcriAr study – São Paulo/Brazil, 2012

The present study used data from ProcriAr study (*The Influence of Nutritional Factors and Urban Air Pollutants on Children’s Respiratory Health: A Cohort Study in Pregnant Women*), which was conducted in the west region of São Paulo – Southeast, Brazil [10, 11]. As lung function in infants was the principal outcome, the sample size was calculated to detect a change of ≥ 5% in pulmonary functional parameters with a study power of ≥ 80% [12], resulting in a required sample size of 400 individuals. Recruitment occurred between March 2011 and December 2013 in four primary health care units. During home visits, all women with positive pregnancy tests who met the eligibility criteria (single fetus, gestational age < 14 weeks and absence of pre-existing chronic diseases) were invited by Community Health Agents to take part of the study. Gestational age was estimated based on the last menstrual period and confirmed by ultrasonography performed in the first trimester of pregnancy. Of the 619 women with positive pregnancy tests, a sample of 454 met the eligibility criteria, provided Informed Consent Form and completed all the questionnaires (Fig. 1).

### Food intake assessment

A validated 110-item quantitative food frequency questionnaire (FFQ) was used to assess the pre-pregnancy food intake of the population [13, 14]. The interviewer-administered FFQ assessed the dietary intake of women for the previous 12 months that is the usual dietary intake prior to becoming pregnant. Frequency of intake of each food item over the pre-pregnancy period and the portion size typically eaten (small, medium or large) were asked during the first home visit, when most of the women were in the first trimester of pregnancy (mean gestational age: 10.7 weeks, range: 6–16 weeks). Foods and recipes listed in the FFQ were converted into grams using Brazilian specific tables and manuals [15]. Daily intakes were calculated by multiplying the portion size by the frequency of intake (1–10) and dividing by the days (daily-1, weekly-7, monthly-30 or yearly-365).

The Nutrition Data System for Research software version 2.0 (2007) (NDSR), developed by the Nutrition Coordinating Centre, University of Minnesota, Minneapolis, MN was used to calculate the dietary intake of energy and nutrients. The nutritional information of the NDSR software is based on data from food composition databases published by the United States Department for Agriculture (USDA). The food contents of NDSR were compared with the Brazilian food composition tables, ensuring that the foods used had an 80–120% match between tables for energy and macronutrients [16].

Daily intake of energy, carbohydrate, protein, fat, alcohol, caffeine, sodium, iron, calcium, vitamin D, docosahexaenoic acid (DHA), dietary folate equivalents (DFE), natural folate, synthetic folate (from fortified foods), methionine, choline, betaine, and vitamins B6 and B12 were analyzed, since those nutrients are part of the nutritional recommendation for a healthy pregnancy [9, 17–20].

### Assessment of socio-demographic and lifestyle factors

The choice of the socio-demographic and lifestyle factors that could influence the dietary pattern of pregnant women was based on previous studies that addressed the determinants of dietary intake among adult population [21, 22], and, also, based on the data collected in the first home visit of the ProcriAr study, through face-to-face interviews. Therefore, age, education, work status, ethnicity, region of birth, relationship status, nutritional status, dietary supplements use, family history of disease (mother or father), smoking habits and sedentary behavior represented the socio-demographic and lifestyle factors considered in this study [21].

The two primary indicators of socioeconomic status (education - as accumulation of schooling - and income) are reportedly associated with one another and also associated with health and disease [23, 24]. Because in ProcriAr study 20% of the participants answered that they did not know or did not want to inform their family income, in this study only education was analyzed as a proxy for socioeconomic status.

Weight (kilograms) and height (meters) were measured according to World Health Organization (WHO) protocol [25]. Body mass index (BMI) was calculated and categorized according to WHO criteria, generating the variable nutritional status [26].

### Statistical analyses

Dietary patterns were estimated using principal component factor analysis and were based on the average daily amount of intake derived from the FFQ food items. Low-fat milk, skim milk, butter/margarine light, unsweetened coffee, unsweetened tea and diet/light soda were consumed by less than 5% of the population and were not included in the analysis. The grouping scheme was based on the correlations between food items and composition similarities, resulting in 51 food items, which were included in the dietary pattern analysis. Food items, grouping description, frequency of intake, and the daily amount of intake for each item were presented in Additional file 1: Table S1.

To identify the number of dietary patterns to be retained, the eigenvalue > 1.0 criterion was used, retaining 17 factors with low interpretability [27]. The Scree test analysis and the interpretation of each factor were considered, resulting in four dietary patterns for further analyses. Varimax orthogonal rotation was performed to simplify the factor matrix and to facilitate data interpretation by generating nonrelated factors. Rotated factor loadings > 0.25 or < − 0.25 were considered to significantly contribute to a pattern [8, 28, 29]. We excluded the items fruit smoothies, sweetened coffee, *farofa*, cassava or corn (flour), offal, pasta with meatless sauce and vinaigrette from the final analysis because they did not load on any of the retained factors. The highest factor loadings were considered when identifying a name for each of the dietary patterns.

The dietary pattern scores were divided into quintiles. Socio-demographic and lifestyle factors were described according to the maternal adherence to a dietary pattern (lower adherence: 1st quintile; higher adherence: 5th quintile). Chi-square tests were used to determine if there were any significant differences between the groups of women classified in the 1st versus 5th quintile for a specific dietary pattern.

Associations between the component scores of each dietary pattern and the covariates were tested in multivariate linear regression models. Age was used in years; underweight, overweight and obese were defined in relation to normal weight using a dummy variable for nutritional status. Northeast and other regions (South, North and Midwest) were defined in relation to Southeast using a dummy variable for region of birth. The following variables were treated as dichotomous (yes or no): eight or more years of education, white skin, dietary supplements use, lacking of formal work, having a partner, no family history of hypertension, no family history of diabetes, not currently smoking and watching two or more hours of TV/day. Each model was adjusted for the other dietary patterns and also for the primary health care unit. The assumed linear relationship between the variables was evaluated using residual plots.

Spearman’s correlation coefficients (r_s_) were calculated for testing associations between the dietary patterns (factors) and the nutrients intake. As the orthogonal rotation of all patterns ensured that they were uncorrelated, the sum of the squared correlations between absolute nutrient availability and the factor scores could be interpreted as the proportion of variance of the nutrient intake explained by the patterns [30]. A radar chart was generated with energy and nutrients represented on the axes to visualize the correlations established with the dietary patterns.

All analyses were performed using Stata Statistical Software (release 12, 2011, StataCorp LP, College Station, TX) [31]. Two-sided significance was determined at *P* < 0.05.

## Results

Four dietary patterns were retained in the principal component factor analysis and accounted for 25.5% of the variance in food intake. The patterns were named ‘Lentils, whole grains and soups,’ ‘Snacks, sandwiches, sweets and soft drinks,’ ‘Seasoned vegetables and lean meats,’ and ‘Sweetened juices, bread and butter, rice and beans’ (Table 1).

**Table 1:** Pre-pregnancy dietary patterns for women, ProcriAr study (*n* = 454) - São Paulo/Brazil, 2012

	Lentils, whole grains and soups	Snacks, sandwiches, sweets and soft drinks	Seasoned vegetables and lean meats	Sweetened juices, bread and butter, rice and beans
Food items^a^	Rotated factor loadings			
Lentils	**0.54**	0.01	0.15	−0.10
Wheat bread and brown rice	**0.51**	−0.10	− 0.03	− 0.04
Soups	**0.47**	0.01	0.21	0.07
Popcorn	**0.47**	0.17	0.04	0.09
Cereal ready to eat and Oats	**0.46**	0.02	0.16	0.04
White cheese	**0.44**	0.07	0.08	−0.01
Desserts with fruits and jelly	**0.44**	0.00	0.23	−0.09
Simple cakes	**0.41**	0.06	0.01	0.13
Soy beverages	**0.41**	−0.02	0.11	0.00
Beef jerky	**0.40**	0.13	−0.06	0.08
Nuts	**0.40**	0.23	0.00	−0.05
Crackers	**0.35**	0.09	−0.01	− 0.03
Soy sauce	**0.35**	0.08	0.10	−0.02
Tea (sweetened)	**0.30**	0.00	0.19	−0.02
Beef (roasted, cooked or soaked)	**0.29**	0.22	0.00	−0.07
Processed meats, sandwiches and snacks	−0.05	**0.59**	0.04	−0.03
Sandwich sauces	0.07	**0.54**	−0.04	−0.09
Desserts and sweets	0.19	**0.50**	−0.05	0.06
Soft drinks	−0.14	**0.48**	−0.16	0.10
Pasta with meat sauce and gnocchi	0.13	**0.44**	0.16	−0.02
Stuffed pasta (cannelloni, lasagne)	**0.32**	**0.39**	0.02	0.05
Yogurt with flavour (whole milk)	0.06	**0.39**	0.03	−0.19
Pork and Frankfurters	−0.01	**0.39**	0.06	0.09
Bakery with filling (cake and cookies)	0.12	**0.38**	−0.09	0.19
Fried beef and fried chicken	−0.09	**0.38**	0.10	0.13
Fried egg or omelette	0.14	**0.36**	0.12	**0.33**
Potato salad, with vegetables and mayonnaise	0.03	**0.36**	**0.27**	0.14
Alcoholic beverages (beer, wine and *caipirinha*^b^)	0.03	**0.35**	−0.02	**−0.29**
Chocolate milk (powder)	0.01	**0.33**	0.03	0.25
*Feijoada* ^c^	**0.31**	**0.32**	0.05	0.12
Potato or cassava (fried)	−0.06	**0.32**	−0.08	**0.30**
Mozzarella cheese	0.22	**0.30**	0.20	−0.04
Vegetables	0.20	−0.05	**0.69**	−0.11
Oil (for salad dressing)	−0.03	0.04	**0.67**	−0.05
Salt	−0.07	0.00	**0.66**	−0.03
Lean meats and fish	0.25	0.00	**0.53**	0.02
Potato or cassava (boiled or roasted)	0.21	0.13	**0.39**	0.06
Fruits	**0.29**	−0.09	**0.31**	−0.07
Sweetened juices (natural or artificial)^d^	0.08	−0.02	−0.13	**0.70**
Butter or margarine (regular and salted)	−0.19	0.17	0.19	**0.46**
French bread and white rice	**−0.27**	0.11	**0.32**	**0.39**
Beans	−0.01	0.09	0.23	**0.36**
Whole milk (3.5–4% fat)	−0.07	0.04	0.16	**0.36**
Yogurt (whole milk)	**0.26**	**−0.32**	0.05	**0.33**
Unsweetened juices (natural or artificial)	0.04	0.04	**0.29**	**−0.59**
Percentage of variance explained (%)	**9.9**	**6.6**	**4.7**	**4.3**

In bold are the rotated factor loadings > 0.25 or < −0.25. ^a^The food items fruit smoothies, sweetened coffee, *farofa*, cassava or corn (flour), offal, pasta with meatless sauce, and vinaigrette were excluded from this analysis because they did not load on any of the retained factors. Traditional recipes: ^b^*Caipirinha* - a drink made with *cachaça* (a hard liquor from sugar cane), fresh limes, sugar and ice; ^c^*Feijoada*: black bean stew. ^d^Natural juices are made with fresh fruits or frozen fruit pulps, with the addition of water or not. Artificial juices are artificial powdered drink mixes, fruit nectars, or sweetened processed juice

The mean age of the women was 26.1 years (Standard deviation (SD) 6.3; range 14–49), and 51% were overweight/obese. Only 10.3% (*n* = 45) had more than 8 years of formal education, the majority was non-white (*n* = 271, 60%), and 30% (*n* = 135) were migrants from outside of the Southeast region. Almost half of them (*n* = 220, 48%) were formally working, and the majority had elementary occupations, such as store attendant, saleswoman, cashier and housekeeper. Forty-one percent (*n* = 186) and 15% (*n* = 68) had a family history of hypertension and diabetes, respectively. Forty-two percent (*n* = 184) spent 2 or more hours/day watching TV and 13% (*n* = 61) were current smokers (Table 2).

**Table 2:** Socio-demographic and lifestyle characteristics of pregnant women according to their dietary patterns, ProcriAr study (*n* = 454) - São Paulo/Brazil, 2012

Characteristics	Total	Lentils, whole grains and soups		Snacks, sandwiches, sweets and soft drinks		Seasoned vegetables and lean meats		Sweetened juices, bread and butter, rice and beans	
		Q1	Q5	Q1	Q5	Q1	Q5	Q1	Q5
	*n* (%)	*n* (%)	*n* (%)	*n* (%)	*n* (%)	*n* (%)	*n* (%)	*n* (%)	*n* (%)
Years of age, mean (SD)	26.1 (6.3)	23.9 (5.7)	29.1 (5.7)***	27.3 (6.8)	25.1 (5.7)*	25.3 (6.7)	26.5 (6.3)	26.8 (5.4)	25.9 (6.6)
Years of formal education									
≤5	111 (24.6)	33 (36.3)	11 (12.2)***	31 (34.1)	18 (20.0)*	26 (28.6)	19 (21.1)	23 (25.3)	29 (32.6)
6–7	96 (21.2)	20 (22.0)	18 (20.0)	24 (26.4)	17 (18.9)	22 (24.2)	14 (15.6)	18 (19.8)	19 (21.4)
≥8	245 (54.2)	38 (41.8)	61 (67.8)	36 (39.6)	55 (61.1)	43 (47.3)	57 (63.3)	50 (55.0)	41 (46.1)
Ethnicity^a^									
*Parda*^b^	216 (47.9)	45 (50.0)	41 (45.6)	49 (53.9)	44 (48.9)	41 (45.1)	45 (50.0)	45 (50.0)	44 (48.9)
White	180 (39.9)	37 (41.1)	39 (43.3)	31 (34.1)	32 (35.6)	39 (42.9)	36 (40.0)	35 (38.9)	40 (44.4)
Black	53 (11.8)	8 (8.9)	8 (8.9)	10 (11.0)	14 (15.6)	10 (11.0)	9 (10.0)	10 (11.1)	6 (6.7)
Other^c^	2 (0.4)	0 (0.0)	2 (2.2)	1 (1.1)	0 (0.0)	1 (1.1)	0 (0)	0 (0.0)	1 (0.0)
Relationship status									
Married or in common law marriage	271 (59.8)	46 (50.6)	60 (67.4)	60 (65.9)	46 (51.1)	45 (49.5)	58 (64.4)*	59 (65.6)	52 (57.8)
Single	178 (39.3)	45 (49.5)	28 (31.5)	30 (33.0)	43 (47.8)	46 (50.6)	30 (33.3)	30 (33.3)	38 (42.2)
Divorced or widower	4 (0.9)	0 (0.0)	1 (1.1)	1 (1.1)	1 (1.1)	0 (0.0)	2 (2.2)	1 (1.1)	0 (0.0)
Region of birth									
Southeast	319 (70.3)	73 (80.2)	57 (63.3)*	47 (51.7)	67 (74.4)**	58 (63.7)	66 (73.3)	59 (64.8)	61 (67.8)
Northeast	122 (26.9)	18 (19.8)	28 (31.1)	42 (46.2)	18 (20.0)	31 (34.1)	19 (21.1)	29 (31.9)	25 (27.8)
Other^d^	13 (2.8)	0 (0.0)	5 (5.6)	2 (2.2)	5 (5.6)	2 (2.2)	5 (5.6)	3 (3.3)	4 (4.4)
Nutritional status									
Underweight	15 (3.3)	3 (3.3)	3 (3.3)	2 (2.2)	4 (4.4)	4 (4.4)	2 (2.2)	1 (1.1)	5 (5.6)*
Normal weight	210 (46.3)	45 (49.5)	34 (37.8)	36 (39.6)	37 (41.1)	45 (49.5)	45 (50.0)	32 (35.2)	46 (51.1)
Overweight	143 (31.5)	30 (33.0)	32 (35.6)	41 (45.1)	30 (33.3)	25 (27.5)	31 (34.4)	34 (37.4)	24 (26.7)
Obese	86 (18.9)	13 (14.3)	21 (23.3)	12 (13.2)	19 (21.1)	17 (18.7)	12 (13.3)	24 (26.4)	15 (16.7)
Dietary supplements use	19 (4.2)	6 (6.6)	3 (3.3)	2 (2.2)	3 (3.3)	3 (3.3)	3 (3.3)	3 (3.3)	3 (3.3)
No formal work	234 (51.5)	57 (62.6)	39 (43.3)	51 (56.0)	35 (38.9)*	40 (44.0)	53 (58.9)*	39 (42.9)	50 (55.6)
Family history of disease (mother or father)									
No family history of hypertension	264 (58.7)	57 (64.0)	47 (52.2)	52 (57.1)	51 (58.0)	55 (61.1)	50 (56.2)	40 (44.0)	58 (64.4)**
No family history of diabetes	383 (84.9)	79 (87.8)	74 (82.2)	75 (82.4)	79 (89.8)	76 (84.4)	75 (83.3)	73 (80.2)	75 (83.3)
Not a current smoker	392 (86.5)	65 (72.2)	85 (94.4)	84 (92.3)	74 (83.2)	81 (90.0)	80 (88.9)	75 (82.4)	79 (87.8)
≥ 2 h/day watching TV	184 (41.6)	46 (52.9)	22 (25.3)	35 (38.9)	38 (43.7)	33 (38.4)	37 (43.0)	40 (44.9)	31 (34.8)
Primary health care unit^e^									
1	185 (40.7)	36 (39.6)	42 (46.7)	42 (46.2)	33 (36.7)	48 (52.7)	32 (35.6)**	33 (36.3)	38 (42.2)
2	213 (46.9)	45 (49.4)	41 (45.5)	39 (42.9)	43 (47.8)	30 (33.0)	50 (55.5)	48 (52.7)	38 (42.2)
3	43 (9.5)	8 (8.8)	5 (5.6)	9 (9.9)	9 (10.0)	9 (9.9)	7 (7.8)	8 (8.8)	13 (14.5)
4	13 (2.9)	2 (2.2)	2 (2.2)	1 (1.1)	5 (5.5)	4 (4.4)	1 (1.1)	2 (2.2)	1 (1.1)

Q1: 1st quintile; Q5: 5th quintile. Chi-square tests were used to determine if there were any significant differences between the 1st and the 5th quintile of each dietary pattern score regards to the women’s characteristics *p < 0.05, **p < 0.01, ***p < 0.001. Missing number of cases for: Years of formal education (2), Ethnicity (3), Relationship status (1), Family history of hypertension (4), Family history of diabetes (3), Smoking (1), and Hours/day watching TV (12). ^a^Based on self-reported skin colour. ^b^*Parda* ethnicity means a mixed-ethnicity, brown skin. ^c^Other in Ethnicity includes Asian and Indigenous population. ^d^Other in Region of Birth includes South, North and Midwest of Brazil. ^e^Located in the District of Butantã, West region of Sao Paulo city

The women who adhered more to the ‘Lentils, whole grains and soups’ dietary pattern were older, had higher level of education and were born outside of the Southeast region, when compared with the women with low adherence to this pattern. Those who adhered more to the ‘Snacks, sandwiches, sweets and soft drinks’ dietary pattern were younger, had higher level of education and were born outside of the Northeast region. Higher adherence to the dietary pattern ‘Seasoned vegetables and lean meats’ was more frequent among those who belonged to a specific primary health care unit, and lower adherence to this pattern was verified among single women and with a formal work. Higher adherence to the ‘Sweetened juices, bread and butter, rice and beans’ dietary pattern was more frequent among underweight or normal weight women and with no family history of hypertension (Table 2).

Age, being born in the South, North or Midwest of Brazil and not being a current smoker were positively associated to the ‘Lentils, whole grains and soups’ score. Higher level of education was positively associated to the ‘Snacks, sandwiches, sweets and soft drinks’ score, while age, lack of formal work and being born in the Northeast region of Brazil were negatively associated to this score. Higher level of education was also positively associated to the ‘Seasoned vegetables and lean meats’ score. Lack of formal work was positively associated to the ‘Sweetened juices, bread and butter, rice and beans’ score, as well as no family history of hypertension. Overweight and obesity were negatively associated to the ‘Sweetened juices, bread and butter, rice and beans’ score (Table 3). The residual plots indicated that the assumed linear relationships between the variables were acceptable for all the four multivariate models (data not presented).

**Table 3:** Association between socio-demographic factors and pre-pregnancy dietary patterns of women, ProcriAr study (*n* = 454) - São Paulo/Brazil, 2012

Socio-demographic and lifestyle characteristics	Lentils, whole grains and soups^a^				Snacks, sandwiches, sweets and soft drinks				Seasoned vegetables and lean meats				Sweetened juices, bread and butter, rice and beans			
	Univariate		Multivariate		Univariate		Multivariate		Univariate		Multivariate		Univariate		Multivariate	
	βi	95%CI	βi	95%CI	βi	95%CI	βi	95%CI	βi	95%CI	βi	95%CI	βi	95%CI	βi	95%CI
Age (years)	0.05	0.03;0.06	**0.04**	**0.03;0.06**	−0.02	−0.04;−0.01	**− 0.02**	**−0.04;− 0.01**	0.02	0.00;0.03	0.01	0.00;0.03	−0.01	−0.03;0.00	− 0.01	− 0.03;0.01
≥ 8 years of education	0.29	0.11;0.47	0.08	− 0.10;0.27	0.26	0.07;0.44	**0.27**	**0.07;0.46**	0.22	0.03;0.40	**0.21**	**0.01;0.41**	−0.07	− 0.25;0.12	− 0.03	− 0.23;0.17
White skin (ethnicity)^b^	0.08	− 0.11;0.26	0.06	− 0.13;0.24	0.02	− 0.17;0.21	0.03	− 0.16;0.22	0.00	−0.19;0.19	− 0.08	− 0.28;0.11	0.07	− 0.12;0.26	0.06	− 0.13;0.25
Having a partner	0.22	0.04;0.41	−0.04	−0.23;0.16	− 0.17	−0.35;0.02	− 0.07	− 0.28;0.14	0.24	0.06;0.43	0.19	−0.02;0.40	− 0.04	− 0.22;0.15	0.10	− 0.11;0.31
Region of birth																
Southeast	1.00	–	1.00	–	1.00	–	1.00	–	1.00	–	1.00	–	1.00	–	1.00	–
Northeast	0.29	0.08;0.49	0.14	−0.07;0.36	− 0.44	− 0.65;−0.24	**− 0.31**	**− 0.53;**−**0.09**	− 0.12	−0.33;0.09	− 0.17	− 0.40;0.06	− 0.10	− 0.31;0.11	− 0.10	−0.33;0.12
Other^c^	0.73	0.18;1.28	**0.55**	**0.03;1.07**	0.24	−0.30;0.79	0.31	−0.24;0.85	0.29	−0.26;0.85	0.22	−0.34;0.79	0.06	−0.50;0.61	− 0.04	− 0.59;0.52
Nutritional status																
Underweight	0.09	−0.43;0.62	−0.04	− 0.55;0.47	0.09	− 0.43;0.61	0.12	− 0.41;0.66	− 0.22	− 0.74;0.31	−0.14	− 0.68;0.41	0.25	− 0.27;0.77	0.13	− 0.41;0.67
Normal weight	1.00	–	1.00	–	1.00	–	1.00	–	1.00	–	1.00	–	1.00	–	1.00	–
Overweight	0.08	−0.14;0.29	− 0.01	− 0.22;0.19	− 0.24	− 0.45;−0.03	− 0.13	− 0.35;0.08	0.08	−0.13;0.30	0.07	−0.16;0.29	− 0.26	− 0.47;−0.05	**− 0.24**	**− 0.46;**−**0.03**
Obese	0.28	0.03;0.53	0.22	−0.02;0.47	0.02	−0.23;0.27	0.02	−0.24;0.28	− 0.08	− 0.33;0.17	− 0.17	− 0.44;0.10	−0.43	− 0.67;−0.18	**− 0.39**	**− 0.65;**−**0.13**
Dietary supplements use	−0.20	− 0.66;0.26	− 0.19	− 0.62;0.24	0.06	− 0.40;0.52	0.18	− 0.27;0.63	0.03	− 0.43;0.49	− 0.05	− 0.52;0.41	0.13	− 0.33;0.59	0.14	− 0.31;0.60
No formal work	−0.21	− 0.39;− 0.03	−0.03	− 0.22;0.17	− 0.24	−0.43;−0.06	**− 0.40**	**− 0.60;**−**0.20**	0.15	− 0.04;0.33	0.19	−0.03;0.40	0.22	0.04;0.40	**0.25**	**0.04;0.46**
No family history HT	−0.16	− 0.35;0.03	− 0.02	− 0.22;0.18	0.04	− 0.15;0.23	− 0.06	−0.26;0.15	− 0.09	−0.28;0.09	− 0.08	− 0.29;0.14	0.34	0.15;0.52	**0.33**	**0.12;0.53**
No family history DM	−0.23	− 0.49;0.03	0.00	− 0.26;0.26	0.13	− 0.12;0.39	0.17	− 0.11;0.44	− 0.04	− 0.30;0.22	− 0.01	−0.29;0.27	0.09	−0.17;0.35	− 0.11	− 0.39;0.16
Not a current smoker	0.49	0.23;0.76	**0.46**	**0.19;0.72**	−0.12	− 0.39;0.15	− 0.14	− 0.41;0.14	− 0.06	− 0.34;0.21	− 0.07	−0.35;0.22	0.17	−0.10;0.44	0.16	−0.12;0.44
≥ 2 h/day of TV	−0.27	− 0.46;−0.09	−0.13	− 0.32;0.07	0.05	− 0.14;0.24	0.16	− 0.04;0.37	0.02	− 0.17;0.21	−0.03	− 0.24;0.18	−0.05	− 0.24;0.14	−0.11	− 0.32;0.10

*95%CI* 95% confidence interval, *DM* Diabetes Mellitus, *HT* Hypertension. Statistically significant β is presented in bold. Missing number of cases for: Years of formal education (2), Ethnicity (based on self-reported skin colour) (3), Relationship status (1), Family history of hypertension (4), Family history of diabetes (3), Smoking (1), and Hours/day watching TV (12). ^a^Each dietary pattern regression model was adjusted by the others and also by the primary health care unit. Interactions not included in this model. ^b^White skin compared to non-white skin (*parda*, black and other ethnicities). ^c^Other in Region of birth includes South, North and Midwest of Brazil

**Fig. 2:**
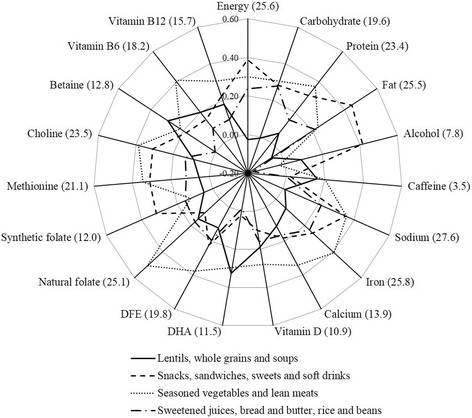
Radar graph of the correlations^a^ (total % of explained variance^b^) between energy and nutrients and the dietary patterns, ProcriAr Study (*n* = 454) - São Paulo/Brazil, 2012. Legends: DHA - docosahexaenoic acid; DFE - dietary folate equivalents. ^a^Correlation coefficients ≥ 0.09 or ≤ − 0.09 were significantly different from 0 (*P* ≤ 0.05). ^b^The variance proportion of energy and nutrient intake explained by the patterns is presented in parentheses

Most of the nutrients investigated were more strongly correlated to ‘Seasoned vegetables and lean meats’ than to the other patterns. In descending order of correlation, these nutrients were natural folate (r_s_ = 0.51), vitamin B6 (0.41), iron (0.41), choline (0.38), DFE (0.38), protein (0.37), sodium (0.36), methionine (0.35), calcium (0.34), vitamin B12 (0.31), carbohydrate (0.30), vitamin D (0.28) and caffeine (0.20). ‘Lentils, whole grains and soups’ had higher correlation coefficients with DHA (0.32) and betaine (0.30). Total fat (0.45) was more strongly associated with ‘Snacks, sandwiches, sweets and soft drinks’, as were alcohol (0.41), energy (0.39) and synthetic folate (0.32). Nevertheless, the four retained patterns accounted for a relatively low proportion of the variance of the studied nutrients, ranging from 27.6% (sodium) to 3.5% (caffeine) (Fig. 2).

## Discussion

This study of women’s pre-pregnancy dietary intake revealed four dietary patterns namely ‘Lentils, whole grains and soups,’ ‘Snacks, sandwiches, sweets and soft drinks,’ ‘Seasoned vegetables and lean meats,’ and ‘Sweetened juices, bread and butter, rice and beans.’ Maternal age, education, work status, region of birth, nutritional status, family history of hypertension and smoking were the factors associated with the dietary patterns. Fruits and vegetables, foods that require preparation, protein, carbohydrate, nutrients related to one-carbon metabolism pathway, DHA, vitamin D, iron, sodium, calcium and caffeine were more strongly correlated to the ‘Seasoned vegetables and lean meats,’ which was the dietary pattern that could contribute most to a healthy pre-pregnancy nutrition [9, 17–20], followed by ‘Lentils, whole grains and soups’ pattern. ‘Snacks, sandwiches, sweets and soft drinks’ pattern was composed of sugar-sweetened and alcoholic beverages, industrialized and takeaway foods, and foods rich in sugar, energy, fat, and synthetic folate, and could be considered an unhealthy dietary pattern [8, 32–35].

Dietary patterns analyses consider the totality of a diet and enables the data collected from observational studies to be translated into descriptions of eating behaviors that can inform public health guidelines and recommendations [36]. In this study, the variance in food intake explained by the dietary patterns was similar to the variance explained by the dietary patterns of a group of 327 pregnant women from the Rio de Janeiro, Brazil (25.3%) [37]. The Brazilian study used an 81-item FFQ to evaluate the dietary intake during pregnancy and identified the following three dietary patterns: ‘healthy’ (legumes, vegetables and fruits), ‘mixed’ (candy, butter and margarine, and snacks) and ‘traditional’ (beans and rice) [37]. The explained variances found in the dietary pattern analyses in both studies have been well accepted in the field of nutritional epidemiology [37–39]. A study review on 54 papers describing maternal dietary patterns and pregnancy outcomes verified that the patterns were frequently classified as prudent or healthy (healthful, health conscious, fruit and low-fat dairy, cooked vegetables, high-protein/fruit, Mediterranean), traditional (common-Brazilian, Nordic, Southern), or as Western or processed (meats/snacks/sweets, high-fat/sugar/takeaway, junk, snack). The majority of the studies identified by the mentioned review study used FFQs to measure the dietary intake and applied principal component analysis to derive the dietary patterns [20].

Higher adherence to dietary patterns consisting of discretionary food items in the pre-pregnancy period has been linked with negative outcomes for both mother and child [20], including maternal uncontrolled asthma [38], gestational diabetes mellitus [39], preterm delivery [40], earlier gestation and shorter birth length [40]. However, these same kind of pre-pregnancy dietary patterns have not been shown to be associated with hypertension [41] nor depressive symptoms [32] in pregnancy, nor with early fetal growth [33] emphasizing the necessity for more studies in this field [34].

Older women appeared to adhere more to a healthy dietary pattern peri-conceptionally and during pregnancy [37, 42]. With increasing age, women from ProcriAr study adhered more to the ‘Lentils, whole grains and soups’ dietary pattern. In contrast, a higher score on the ‘Snacks, sandwiches, sweets and soft drinks’ pattern was associated with being younger. Despite the low levels of education reported by this population, higher education was associated to the dietary patterns ‘Snacks, sandwiches, sweets and soft drinks’ and ‘Seasoned vegetables and lean meats’, which are patterns that included more expensive foods. Studies in Brazil have shown that the proportion of consumption of food groups such as milk and dairy, fruits and vegetables, animal fats, processed meats, alcoholic beverages, soft drinks and ready meals tends to increase consistently with the level of household income, demonstrating the mixed effects of education and income in determining food intake [43, 44].

Migrants typically move to achieve better living conditions, but in Brazil important socioeconomic differences persist when migrants are compared with the native population [45] and are likely to reflect in dietary behavior [46]. Brazil is geopolitically divided into five regions: North, Northeast, Central-West, Southeast and South. The Southeast and South regions are the first and second economies of Brazil, followed by Central-West, Northeast, and North. São Paulo is part of the Southeast region of Brazil, and is considered the most populous city with the largest economy by gross domestic products in the Southern Hemisphere. Almost 30% of the population in this study was not born in Southeast Brazil. Being born in the South, North or Midwest of Brazil was positively related to ‘Lentils, whole grains and soups’ pattern. In contrast, being born in the Northeast region of Brazil was negatively related to the ‘Snacks, sandwiches, sweets and soft drinks’ pattern, demonstrating the poorer socioeconomic conditions and/or the persistence of eating habits acquired in one’s region of birth. In fact, previous study has identified that, for all nine states of the Northeast region of Brazil, the prevalence of fruits and vegetables intake was below the observed national prevalence (average of 26% in Northeast versus 37% in Brazil – including Northeast) [47].

Unemployment was associated with a dietary pattern composed mostly by foods that require preparation (‘Sweetened juices, bread and butter, rice and beans’). In contrast, employment was related to the pattern rich in fast foods and takeaway foods (‘Snacks, sandwiches, sweets and soft drinks’). Being employed is an important determinant of food-related decisions [48]. Van der Horst & Siegrist [48] found correlations between cooking and working status, with workers spending less time cooking and reporting fewer cooking skills. In ProcriAr study, despite of the fact that the unemployed women cooked more than those who were employed, 61% of them watched TV for 2 or more hours/day. In contrast, only 22% of the women formally working had the same sedentary behavior (Chi-square test, *p* < 0.001, unpublished results).

Socio-demographic and lifestyle factors were determinants of the women’s pre-pregnancy dietary patterns in this study, suggesting that extra resources may be necessary for disadvantaged mothers to ensure good nutrition during pregnancy [49, 50]. These results highlight that attention should be prioritized to young employed women of low socioeconomic status, and who are born in urban and highly industrialized regions. Women with these characteristics were more likely to have an unhealthy dietary habit [44, 51, 52], and this knowledge should be considered in an individualized antenatal care.

The Brazilian health system consists of a range of public and private organizations, and people can use both depending on ease of access or their ability to pay [53]. Therefore, socioeconomic inequalities exist between individuals that use the public or the private sectors [53]. Those women who have their antenatal care in the public health service are more likely to be poor, when compared with those who have their antenatal care in the private sector [54]. The women in this study were recruited from public primary health care units, and represent a population from a lower socioeconomic area, with a low level of education and the majority of whom had elementary occupations. This population characteristic can be observed in several pre-conceptional women’s settings in Brazil, and the evidence of this study may be applicable to those settings [54]. The coverage of antenatal care in Brazil is high, and the majority of visits are made to public primary health care units (89.6%) [55]. This represents a window of opportunity to interventions in food and nutrition, since lifestyle characteristics are prone to change in association with pregnancy [20, 55]. A special focus on diet within the antenatal care framework could have a greater impact on maternal and child health, and at a lower cost, than strategies based on postnatal therapy to those with health issues that occurred as a consequence of the pregnancy [56].

Individuals and environmental interventions could be implemented during the antenatal care [7, 57], such as encouraging the involvement of the whole family in meals planning and preparation [58], understanding which are the barriers for cooking and eating more fruit and vegetables (working with participative troubleshooting models) [59], encouraging the intake of seasonal fruit and vegetables, implementing garden-based fruit and vegetables intervention, negotiating the establishment of food markets with local producers [60, 61], and offering culinary workshops [62]. However, to be more effective, nutritional interventions should approach the complex set of dietary behaviors determinants, such as women’s social and material resources, social and cultural environment, psychosocial factors, and accessibility of food [63–65].

This study highlights the relevance and application of the investigation of food patterns and their association with socio-demographic factors during the peri-conceptional period. Additionally, the topic of dietary patterns is a growing area of research that is relevant to nutrition policies and programs [8]. However, the use of factor analysis has been criticized for its subjective nature, including the consolidation of food items into food groups, the number of factors to be extracted and the methods of rotation and labelling [8]. In order to improve the assessment, interpretability and comparability of our results, we have addressed in this manuscript all the items suggested by the STROBE guidelines [66]. A potential limitation of this study is the use of a FFQ as a tool to evaluate the dietary intake, which is a method that rely on memory and recall bias are more likely to occur. The use of foreign food composition tables to estimate the population dietary intake can be also considered a limitation of this study. Unfortunately, the Brazilian food composition tables have few nutrients analyzed, which makes it difficult to use them in dietary intake studies. Yet, the lack of detailed information about the dietary supplements taken (such as brands and quantities) could be underestimating the dietary intake of micronutrients. However, the use of dietary supplements was very low in this population and was not associated with any of the dietary patterns. Still, dietary intake is the product of different factors and interactions, many of which could not be accomplished in this study. Thus, future research are needed in order to fully investigate the relationship between the social determinants of health and women’s dietary patterns [67]. Although ProcriAr study was a cohort study, our study used a cross-sectional analysis. A subsequent research is planned to investigate whether the maternal dietary patterns identified here are related to better or worse outcomes in pregnancy and in the children’s health, both in utero and later in life.

## Conclusions

The dietary pattern analysis led to a better understanding of the pre-pregnancy eating behaviors and their determinant factors among women of childbearing age in ProcriAr study. The analysis of pre-pregnancy food intake produced four distinctive dietary patterns. The ‘Snacks, sandwiches, sweets and soft drinks’ dietary pattern (composed of sugar-sweetened and alcoholic beverages, industrialized and takeaway foods, and foods rich in sugar, energy, fat, and synthetic folate) was associated with being younger, more educated, formally employed and born in the Southeast region of Brazil. Based on its food and drink contents, this dietary pattern could be considered the unhealthiest eating behavior for pregnancy. As women’s health is a public health priority, the findings of this study add perspectives to be considered in the implementation of health promotion practices and interventions that will enable the improvement of women’s nutritional status and provide an adequate environment for a healthy fetal development.

## Additional file

Additional file 1: **Table S1.** Food items, grouping description, frequency of intake and daily amount of intake of women in the pre-pregnancy period, ProcriAr Study (*n* = 454) - São Paulo/Brazil, 2012. (DOCX 26 kb)

## Abbreviations

95% CI: 95% Confidence interval
BMI: Body mass index
DFE: Dietary folate equivalents
DHA: Docosahexaenoic acid
DM: Diabetes mellitus
DP: Dietary patterns
FFQ: Food frequency questionnaire
HT: Hypertension
NDSR: Nutrition data system for research software
Q1: 1st quintile
Q5: 5th quintile
r_s_: Spearman’s correlation coefficients
SD: Standard deviation
USDA: United States Department for Agriculture
WHO: World Health Organization
