# Supplementary material for: Dietary patterns are influenced by socio-demographic conditions of women in childbearing age: a cohort study of pregnant women
Source: BMC Public Health. 2018 Mar 1;18:301. doi: 10.1186/s12889-018-5184-4 (PMC5831579; doi:10.1186/s12889-018-5184-4)
Supplement: Supplementary file 1 — Table S1. Food items, grouping description, frequency of intake and daily amount of intake of women in the pre-pregnancy period, ProcriAr Study (n = 454) - São Paulo/Brazil, 2012. (DOCX 26 kb) [file 12889_2018_5184_MOESM1_ESM.docx]

| **Additional file 1: Table S1.** Food items, grouping description, frequency of intake and daily amount of intake of women in the pre-pregnancy period, ProcriAr Study (n=454) - São Paulo/Brazil, 2012. | | | | |
| --- | --- | --- | --- | --- |
| **Food items** | **Grouping description** | **Intake (%)** | **Daily amount of intake (g/day)** | |
|  |  |  | **Median** | **95% CI** |
| Whole milk (3.5-4% fat) | FFQ | 80.0 | 128.9 | 113.2;128.9 |
| Fruit smoothie (whole milk) | FFQ | 62.1 | 8.3 | 8.3;16.6 |
| Yogurt (whole milk) | FFQ | 25.5 | 0.0 | 0.0;0.0 |
| Yogurt with flavour (whole milk) | FFQ | 62.8 | 14.2 | 11.0;23.7 |
| Cereal ready to eat and Oats | ‘Cereal ready to eat’, ‘oats’ | 44.5 | 0.0 | 0.0;0.0 |
| Mozzarella cheese | FFQ | 74.2 | 5.7 | 5.7;5.7 |
| White cheese | FFQ | 38.1 | 0.0 | 0.0;0.0 |
| Crackers | FFQ | 80.6 | 10.8 | 10.8;11.1 |
| Simple cakes | FFQ | 72.5 | 6.7 | 4.0;8.6 |
| Bakery with filling (cake and cookies) | ‘Cake with filling’, ‘cookies with filling’ | 85.2 | 8.6 | 7.5;10.2 |
| Butter or margarine (regular and salted) | FFQ | 89.9 | 11.6 | 7.7;13.5 |
| Coffee (sweetened) | FFQ | 66.7 | 81.1 | 60.1;81.1 |
| Tea (sweetened) | FFQ | 44.9 | 0.0 | 0.0;0.0 |
| Chocolate milk (powder) | FFQ | 66.3 | 3.6 | 3.3;3.6 |
| Wheat bread and brown rice | ‘Wheat bread’, ‘brown rice’ | 26.9 | 0.0 | 0.0;0.0 |
| French bread and white rice | ‘French bread’, ‘white rice’ | 100.0 | 269.4 | 249.2;286.0 |
| Potato or cassava (boiled or roasted) | FFQ | 91.0 | 20.0 | 18.4;20.0 |
| Potato or cassava (fried) | FFQ | 81.5 | 34.0 | 34.0;34.0 |
| *Farofa*^a^, cassava or corn (flour) | FFQ | 61.2 | 2.5 | 1.1;3.6 |
| Potato salad, with vegetables and mayonnaise | FFQ | 73.6 | 3.0 | 3.0;4.9 |
| Beans | FFQ | 94.9 | 104.8 | 86.0;104.8 |
| Lentils | FFQ | 28.8 | 0.0 | 0.0;0.0 |
| *Feijoada*^b^ | FFQ | 66.3 | 7.0 | 3.1;7.0 |
| Lean meats and fish | ‘Beef with vegetables’, ‘roasted, cooked or soaked chicken’, ‘boiled egg’, ‘roasted, cooked or soaked fish’, ‘fried fish’ | 98.9 | 61.3 | 56.3;66.8 |
| Beef (roasted, cooked or soaked) | FFQ | 85.7 | 24.5 | 17.1;28.6 |
| Pork and Frankfurters | ‘Bacon’, ‘pork sausage’, ‘frankfurters’, ‘pork (loin and chops)’ | 94.9 | 23.6 | 22.2;28.1 |
| Fried beef and fried chicken | ‘Fried beef’, ‘fried chicken’ | 92.1 | 49.3 | 41.1;56.0 |
| Fried egg or omelette | FFQ | 76.4 | 7.1 | 7.1;7.1 |
| Beef jerky | FFQ | 33.9 | 0.0 | 0.0;0.0 |

| **Additional file 1: Table S1.** (continue) | | | | |
| --- | --- | --- | --- | --- |
| **Food items** | **Grouping description** | **Intake (%)** | **Daily amount of intake (g/day)** | |
|  |  |  | **Median** | **95% CI** |
| Offal (heart and liver) | FFQ | 33.5 | 0.0 | 0.0;0.0 |
| Pasta with meat sauce and gnocchi | FFQ | 75.1 | 11.0 | 7.3;12.1 |
| Stuffed pasta (cannelloni, lasagne) | FFQ | 78.8 | 4.8 | 4.8;6.3 |
| Pasta with meatless sauce | FFQ | 78.4 | 28.6 | 20.0;28.6 |
| Soups | ‘Cream soup’, ‘vegetable soup’ | 70.9 | 12.6 | 7.0;14.1 |
| Fruits | ‘Orange’, ‘banana’, ‘apple’, ‘papaya’, ‘melon’, ‘pineapple’, ‘mango’, ‘avocado’, ‘guava’, ‘persimmon’, ‘grape’ | 99.8 | 304.8 | 279.0;325.7 |
| Vegetables | ‘Lettuce’, ‘spinach’, ‘watercress’, ‘kale’, ‘cabbage’, ‘cauliflower’, ‘tomato’, ‘carrot’, ‘eggplant’, ‘beets’, ‘chayote’, ‘pumpkin’, ‘cucumber’, ‘zucchini’, ‘onion’ | 98.9 | 106.7 | 99.0;115.8 |
| Oil (for salad dressing) | FFQ | 89.6 | 3.3 | 3.3;4.6 |
| Salt | FFQ | 94.5 | 0.4 | 0.3;0.4 |
| Soy sauce | FFQ | 17.8 | 0.0 | 0.0;0.0 |
| Vinaigrette | FFQ | 43.6 | 0.0 | 0.0;0.0 |
| Processed meat, sandwiches and snacks | ‘Hamburger/chicken nuggets/meatball’, ‘ham/mortadella/salami’, ‘sandwich: hot dog/hamburger’, ‘fried snacks’, ‘baked savoury’, ‘pizza’, ‘snacks’ | 99.1 | 69.3 | 62.0;75.8 |
| Sandwich sauces | ‘Mayonnaise’, ‘ketchup/mustard’ | 62.1 | 0.6 | 0.4;0.9 |
| Popcorn | FFQ | 59.5 | 0.7 | 0.7;1.1 |
| Nuts | FFQ | 36.3 | 0.0 | 0.0;0.0 |
| Desserts with fruits and jelly | ‘Desserts with fruits’, ‘jelly’ | 70.5 | 5.0 | 3.7;7.0 |
| Desserts and sweets | ‘Candy/lollipop’, ‘whipped cream/coconut milk/condensed milk’, ‘chocolate’, ‘ice cream’, ‘sweet pies/pudding/mousse’ | 96.5 | 34.6 | 30.1;38.6 |
| Soy beverages | FFQ | 33.9 | 0.0 | 0.0;0.0 |
| Soft drinks | FFQ | 81.9 | 59.3 | 52.4;71.9 |
| Sweetened juices (natural^d^ or artificial^e^) | ‘Artificial juice (sweetened)’, ‘natural juice (sweetened)’ | 88.1 | 51.1 | 42.5;60.1 |
| Unsweetened juices (natural or artificial) | ‘Artificial juice (unsweetened)’, ‘natural juice (unsweetened)’ | 32.6 | 0.0 | 0.0;0.0 |
| Alcoholic beverages (beer, wine and *caipirinha*^c^) | ‘Beer’, ‘wine’, *‘caipirinha’* | 41.4 | 0.0 | 0.0;0.0 |
| 95%CI - 95% confidence interval. Traditional recipes: ^a^*Farofa*: manioc flour toasted in butter or olive oil/cooking oil, sometimes mixed with meat or eggs; ^b^*Feijoada*: black bean stew; ^c^*Caipirinha* - a drink made with “cachaça” (a hard liquor from sugar cane), fresh limes, sugar and ice. ^d^Natural juices are made with fresh fruits or frozen fruit pulps, with the addition of water or not. ^e^Artificial juices are artificial powdered drink mixes, fruit nectars, or sweetened processed juice. | | | | |
